# Two cases of MACS due to PBMAH associated with an *in vivo* aberrant response to LHRH treated with leuprolide acetate

**DOI:** 10.1530/EDM-24-0074

**Published:** 2025-11-06

**Authors:** Laurie Branchaud-Croisetière, Michel Maillet, Matthieu St-Jean

**Affiliations:** Division of Endocrinology, Department of Medicine, Research Center, Centre Hospitalier de l’Université de Sherbrooke (CRCHUS), Sherbrooke, Quebec, Canada

**Keywords:** MACS, leuprolide, hypercortisolism, PBMAH, aberrant receptors

## Abstract

**Summary:**

We describe two rare cases of primary bilateral macronodular adrenal hyperplasia (PBMAH) with mild autonomous cortisol secretion (MACS), incidentally discovered and further evaluated for aberrant cortisol responses to various stimuli. In both patients, morning serum cortisol levels remained elevated following 1 mg dexamethasone suppression test (DST) and 4 mg intravenous DST, while basal adrenocorticotropic hormone (ACTH) levels were suppressed. However, 24 h urinary free cortisol and nocturnal salivary cortisol levels were within normal limits. The first case involved a 69-year-old woman who exhibited significant increases in cortisol in response to luteinizing hormone-releasing hormone (LHRH; +142%), vasopressin (+66%) and metoclopramide (+98%). Treatment with the long-acting GnRH agonist leuprolide acetate led to decreased cortisol production and normalization of ACTH levels. The second case, a 54-year-old woman, showed cortisol increases following stimulation with LHRH (+58%), cosyntropin (+1,016%) and vasopressin (+46%). However, leuprolide acetate treatment did not successfully control her hypercortisolism. These cases highlight the clinical relevance of identifying aberrant cortisol responses to specific stimuli in patients with PBMAH and MACS. Such findings may inform the use of targeted medical therapies as alternatives to unilateral or bilateral adrenalectomy. In addition, a more pronounced cortisol response to LHRH compared to other stimuli may predict a favorable response to GnRH agonist therapy.

**Learning points:**

- Systematic dynamic stimulation testing to assess cortisol response to a variety of stimuli should be offered to all patients with primary bilateral adrenal hyperplasia (PBMAH) with indication for treatment.
- Identification of an aberrant response suggesting potential overexpression of *β*-adrenergic or luteinizing hormone/chorionic gonadotropin receptors (LHCGRs) may allow the use of targeted medical therapies, instead of unilateral or bilateral adrenalectomy, to control the cortisol excess in a subset of patients.
- In patients with MACS who have an aberrant cortisol response following LHRH stimulation, the long-acting GnRH agonist leuprolide acetate can be used to suppress endogenous LH production and improve hypercortisolism.
- A greater increase in cortisol secretion following LHRH stimulation and small response to the other stimuli could potentially be predictive of a better response to treatment with leuprolide acetate.

## Background

MACS can be defined by a serum cortisol >50 nmol/L after 1 mg DST and the absence of overt clinical stigmata of Cushing’s syndrome (1, 2). PBMAH is a rare cause of adrenal CS accounting for <2% of cases (3). Several factors have been implicated in the pathogenesis of PBMAH, including pathogenic genetic variants, paracrine production of ACTH and aberrant expression of eutopic or ectopic G-protein coupled receptors (GPCRs) on the adrenal glands of these patients (3, 4). Aberrant receptors that have been described in adrenal glands of patients with CS and MACS patients include those for vasopressin (AVPR1, AVPR2, and AVPR3), serotonin (HTR4), luteinizing hormone/chorionic gonadotropin (LHCGR), *β*-adrenergic receptors (ADRBs), glucose-dependent insulinotropic peptide (GIPR), angiotensin II (AT1R), glucagon (GCGR) and serotonin (HTR7) (4). According to five studies that systematically screened patients with overt or mild adrenal CS for aberrant GPCR expression, 80% showed an exaggerated cortisol response to at least one stimulus and 50% showed multiple responses, some with up to four aberrant responses (4). The most common aberrant responses *in vivo* were to vasopressin and 5HT4R agonists (4). Identification of an abnormal response to one of these stimuli, suggesting the presence of functional aberrant receptors on the adrenal glands, may allow the use of targeted medical therapies as an alternative to adrenalectomy in selected patients. Few cases of hypercortisolism due to aberrant LHCGRs have been reported in the literature, only four of which were treated with GnRH agonists (4). In the present article, we describe two rare cases of patients with mild cortisol secretion due to PBMAH and an exaggerated cortisol increment following *in vivo* LHRH stimulation. The first patient had a significant biochemical response to leuprolide acetate, whereas the second patient failed to respond to treatment after 10 months.

## Case presentation

### First case

A 69-year-old woman was evaluated in 2020 for PBMAH that was incidentally discovered on thoracic imaging. Her past medical history included thoracic aortic aneurysm, depression, insomnia and osteoporosis associated with multiple fragility fractures. She was an active smoker and was taking citalopram, calcium and vitamin D. In 2021, she was diagnosed with dyslipidemia and hypertension and had a new osteoporotic fracture. She was subsequently started on atorvastatin, irbesartan and alendronate. On physical examination, there were no classic signs of hypercortisolism.

## Investigation

### Laboratory and imaging findings

Initial endocrine evaluation was performed between 2020 and 2021 (Table 1).

**Table 1:** Case 1: biochemical endocrine evaluation initially and after treatment with leuprolide acetate.

Test	Initial evaluation 2020–2021	1–8 months following treatment	9–14 months following treatment	Normal range
1 mg PO DST (nmol/L)^†^	133	-	104	<50
4 mg IV DST (nmol/L)	185 (day 2)	-	-	<130
Late-night salivary cortisol (nmol/L)^†^	<3	-	4	<5
ACTH (pmol/L)^†^	1.7	2.7	5.2	
DHEAS (umol/L)^†^	1.6	0.66	1.4	0.95–11.67
PAC (pmol/L)	79	-	-	<350
Renin mass (ng/L)	1.4	-	-	3–16
PAC-to-renin ratio (pmol*L/ng*)	56	-	-	<60
17-OHP (nmol/L)	<0.3	-	-	0.5–2.8
HbA1C (%)^†^	5.6	5.7	5.8	4–6
TC (mmol/L)^†^	6.44	4.27	4.11	-
Triglycerides (mmol/L)^†^	1.42	1.26	1.14	-
C-LDL (mmol/L)^†^	4.73	2.29	2.01	-
C-HDL (mmol/L)	1.48	1.8	1.77	-
FSH (UI/L)	85.5	22	-	20–138
LH (UI/L)	30.4	0.6	-	10.4–64.6

DST, dexamethasone suppression test; PAC, plasmatic aldosterone concentration; TC, total cholesterol.

* Atorvastatin was started in 2021.

^†^ Highest value shown.

Her worsening comorbidities and abnormal 1 mg DST prompted dynamic stimulation testing to assess cortisol responses to various stimuli, which was carried out in February 2022 (Table 3). During these studies, dexamethasone 1 mg PO every 6 h was administered concomitantly to ensure ACTH suppression throughout the tests. A post-stimulus cortisol increase was considered significant if it exceeded 50% above baseline. She demonstrated an exaggerated cortisol response to LHRH (+142%), vasopressin (+66%) and metoclopramide (+98%), but not to other tested stimuli (Table 3). Due to sample handling problems, cortisol measurements following stimulation with cosyntropin and the mixed-meal test were not performed. Genetic testing for pathogenic variants in *ARMC5* and *KDM1A* was negative. No pathological evaluation was performed to confirm the presence of aberrant receptors, as the patient did not undergo unilateral adrenalectomy (UA).

**Figure 1:**
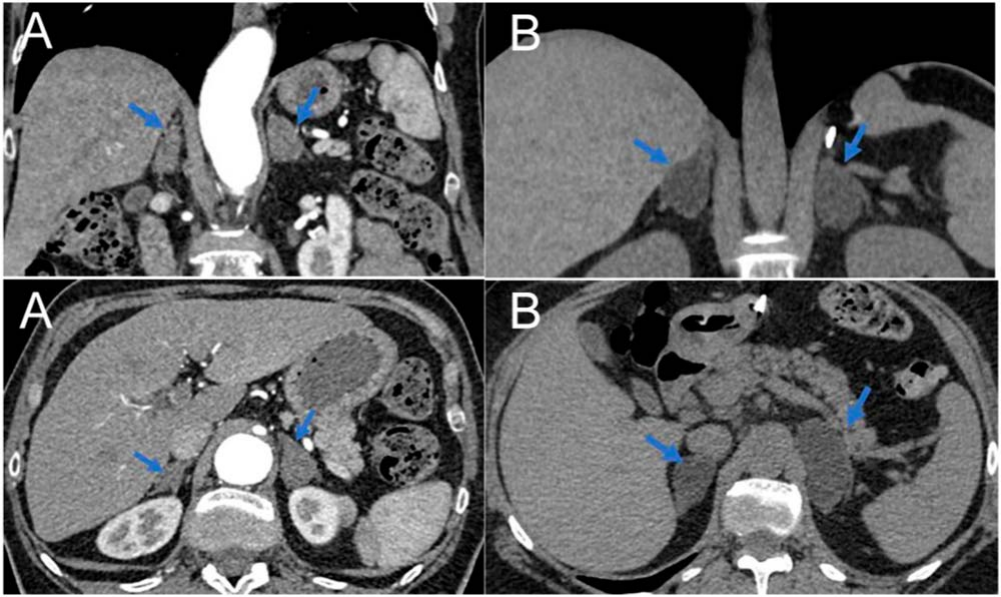
Abdominal CT scan with IV contrast. Coronal and axial images of the abdomen showing PBMAH with a right adrenal nodule of 35 × 15 mm and a left adrenal nodule of 20 × 27 mm for case 1 (A) and PBMAH with a right adrenal nodule of 22 × 28 mm and a left adrenal nodule of 24 × 50 mm for case 2 (B).

In March 2020, a non-contrast thoracic CT scan revealed a right adrenal nodule measuring 3 × 1.4 cm and a left adrenal nodule measuring 3 × 2 cm, both with densities <10 HU, along with hypertrophy of the adrenal limbs. The size of the nodules remained stable on a contrast-enhanced thoracoabdominal CT scan performed in February 2022 (Fig. 1).

In August 2021, an FDG PET scan showed a maximum SUV of 3.4 on the right and 3.7 on the left.

## Treatment

**Figure 2:**
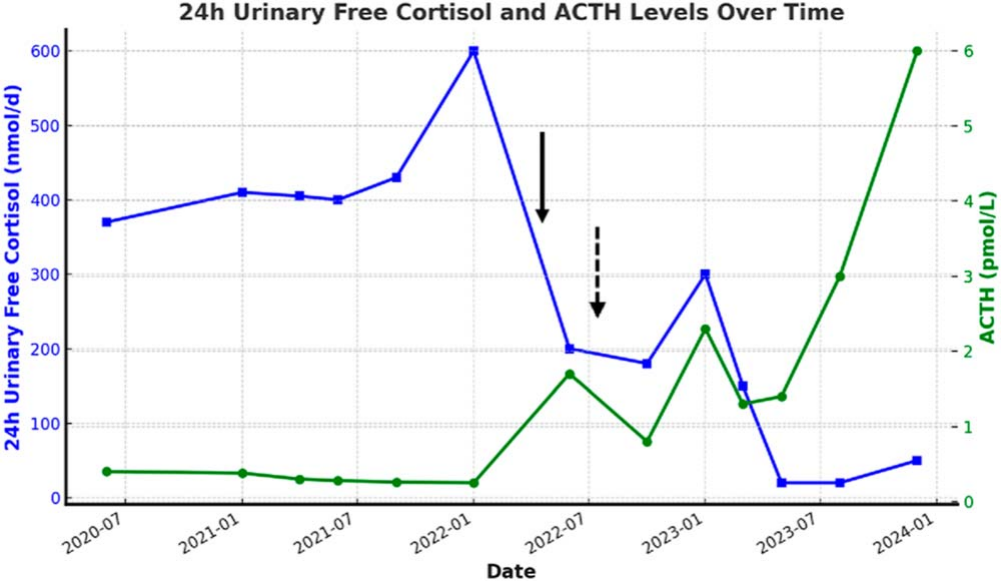
24 h Urinary cortisol excretion (UFC) and ACTH levels before and after treatment with leuprolide acetate for the first patient. The first arrow indicates introduction of leuprolide 3.75 mg IM monthly, and the second dotted arrow indicates when the dose was increased to 7.5 mg IM monthly. To convert values in μg/24 h, divide by 2.76.

The long-acting GnRH agonist leuprolide 3.75 mg IM every four weeks was introduced in April 2022 to suppress endogenous LH production and was associated with a partial response. The dose was subsequently titrated to 7.5 mg IM every four weeks in August 2022. After dose titration, the 24 h UFC decreased slightly and ACTH increased from <1.1 at baseline to 5.2 pmol/L in June 2023 (Fig. 2). Repeated 1 mg DST did not normalize despite effectively suppressed LH and FSH levels by leuprolide (Table 1).

## Case presentation

### Second case

A 54-year-old woman was referred in 2022 for suspected CS and PBMAH detected on lumbar CT scan in 2019. She had been diagnosed with hypercholesterolemia, anxiety and obesity (maximum BMI of 40 kg/m^2^ in 2015). She underwent gastric sleeve bariatric surgery in 2016 and subsequently lost 30 kg, but she had regained 15 kg between 2017 and 2022. She was taking atorvastatin 20 mg once daily and semaglutide 1 mg SC weekly, which was added for weight control in 2023. Her weight remained stable during subsequent follow-ups. On physical examination, she had increased cervical adipose tissue and mild supraclavicular fat pads but no other physical signs suggestive of a classic CS phenotype.

## Investigation

### Laboratory and imaging findings

Initial endocrine evaluation was performed between 2020 and 2023 (Table 2).

**Table 2:** Case 2: biochemical endocrine evaluation initially and after treatment with leuprolide acetate.

Test	Before treatment 2020–2023	1–6 months following treatment	7–10 months following treatment	Normal range
1 mg PO DST (nmol/L)	157; 107; 140	-	88	<30
4 mg IV DST (nmol/L)	183 (day 2)	-	-	<130
Late-night salivary cortisol (nmol/L)	<2–4 (4 values)	<3 (3 values)	<3 (1 value)	<3
ACTH (pmol/L)	<1.1–1.3 (3 values)	<1.1–1.4 (5 values)	<1.1, <1.1	0–10
DHEAS (umol/L)	0.7, 0.8, 0.9	0.7–1.1 (6 values)	0.9–1 (2 values)	0.9–1.5
17-OHP (nmol/L)	0.62	-	0.5	0–6
HbA1C (%)	5.6, 5.7	-	-	4–6
TC (mmol/L)	3.6–4.86 (4 values)	-	4.39	-
Triglycerides (mmol/L)	0.88–1.62 (4 values)	-	1.47	-
C-LDL (mmol/L)	1.58–2.73 (4 values)	-	2.23	-
C-HDL (mmol/L)	1.41–1.73 (4 values)	-	1.48	-
FSH (UI/L)	83.8	4.4–6 (4 values)	5–5.5	26–135
LH (UI/L)	44.6	<0.3–0.67 (4 values)	<0.3, <0.3	8–59

DST, dexamethasone suppression test; TC, total cholesterol.

Dynamic stimulation testing under dexamethasone suppression (1 mg PO every 6 h) was also performed to assess cortisol responses to a variety of stimuli (Table 3). She had an increase in cortisol following LHRH (+58%), vasopressin (+328%) and cosyntropin 250 μg IV (+1,016%). No significant increases (>50%) in cortisol were observed during other tests. Due to sample handling problems, cortisol measurements after stimulation with glucagon 1 mg IV were not obtained. Genetic testing for pathogenic variants in *AMRC5* and *KDM1A* was negative.

**Table 3:** *In vivo* cortisol response following the stimulation with several stimuli. ACTH levels (not shown) remained suppressed (<1.1 pmol/L; 5 pg/mL) throughout the study. An increase in cortisol level of >50% following stimulation is considered a significant response. Bold text is used to highlight the response that is considered to be a significantly abnormal response to the stimuli.

Test	Patient 1			Patient 2		
	Serum cortisol at baseline (nmol/L)	Peak serum cortisol (nmol/L)	Relative cortisol increase from baseline value (%)	Serum cortisol at baseline (nmol/L)	Peak serum cortisol (nmol/L)	Relative cortisol increase from baseline value (%)
Upright posture	131	121	−8	100	118	18
Mixed meal	122	N/A	N/A	320	287	−10
Cosyntropin 250 μg IV	N/A	N/A	N/A	**127**	**1,418**	**1,016**
Vasopressin 10 mg IM	**127**	**211**	**66**	**105**	**449**	**328**
LHRH 100 μg IV	**151**	**365**	**142**	**98**	**155**	**58**
Glucagon 1 mg IV	104	106	2	N/A	98	N/A
Metoclopramide 10 mg PO	**110**	**218**	**98**	96	140	46

LHRH, luteinizing hormone-releasing hormone; N/A, not available.

To convert serum cortisol values to μg/dL, divide by 27.6.

See Fig. 2 for abdominal CT scan images.

## Treatment

**Figure 3:**
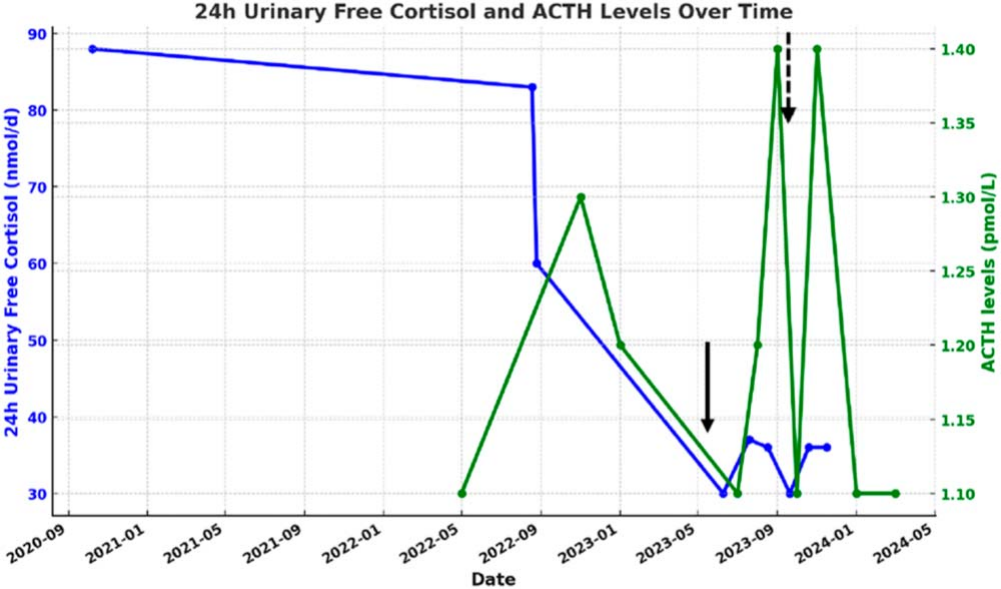
24 h Urinary cortisol excretion (UFC) and ACTH levels before and after treatment with leuprolide acetate for the second patient. The first arrow indicates introduction of leuprolide acetate 3.75 mg IM every four weeks, and the second dotted arrow indicates when the dose was increased to 7.5 mg IM every four weeks. To convert ACTH levels in pg/mL, divide by 0.22.

Leuprolide 3.75 mg IM every four weeks was started in June 2023. Initially, there was a slight increase in ACTH levels, and therefore, the dose was increased to 7.5 mg IM every four weeks in November 2023. Despite dose titration, ACTH levels remained suppressed throughout the follow-up (Fig. 3). 1 mg DST decreased but did not normalize on repeat dosing (Table 2), and 24 h UFC remained in the normal range without significant variation (Fig. 3). LH and FSH levels were effectively suppressed by leuprolide (Table 2). Given the lack of response after 10 months of therapy, the patient had a unilateral left adrenalectomy. The expression of aberrant LH receptors was almost absent on pathological evaluation.

### Other previously reported cases in the literature

Lacroix *et al.* (5) reported the first patient with aberrant cortisol elevation following LHRH stimulation, who was a 68-year-old woman with transient clinical CS during successive pregnancies and eventually developed postmenopausal persistent CS and PBMAH. She had an aberrant cortisol response to gonadotropin-releasing hormone (GnRH) (+214%) and human chorionic gonadotropin (+197%). Long-term treatment with leuprolide 3.75 mg IM monthly resulted in reversal of CS (Table 4). Two other cases of MACS and CS due to PBMAH associated with an exaggerated cortisol response after LHRH stimulation (+184% and +>150%, respectively) were treated with leuprolide and showed significant improvements (6, 7). Bovenberg *et al.* (8) described a case of adrenal CS in a postmenopausal woman with PBMAH and an exaggerated response to LHRH (+565%) *in vivo*. Administration of leuprolide resulted in a transient improvement of hypercortisolism, but an escape from treatment was documented after eight months of treatment and bilateral adrenalectomy was eventually performed. *In vitro* studies of the adrenal cells showed an aberrant exaggerated response to ACTH and metoclopramide but not to LH. In addition, the mRNA expression of the LH receptor was low in the patient’s adrenal gland. Thus, it can be hypothesized that this patient may have lost her response to LH due to downregulation of the LHCGRs on the tumor cells.

**Table 4:** Reported cases of patients with overt CS or MACS and aberrant regulation of cortisol secretion following LHRH stimulation treated with leuprolide acetate, adapted from St-Jean *et al.* (4).

Characteristics	Lacroix *et al.* (5)	Bourdeaux *et al.* (6)	Bovenberg *et al.* (8)	Karapanou *et al.* (7)	Present study	
					Case 1	Case 2
Sex	Female	Female	Female	Female	Female	Female
Age, years	63	51	48	57	69	54
CS or MACS	CS	CS	CS	MACS	MACS	MACS
BMAH or unilateral adenoma	BMAH	BMAH	BMAH	BMAH	BMAH	BMAH
Aberrant response in vivo*						
GNRH	+214%	+184%	+143%			
hCG	+197%			>50%		
LHRH			+565%	>50%	+142%	+58%
Other significant aberrant responses in vivo*				None		
ACTH	+807%					+1,016%
Metoclopramide	+157%				+98%	
Cisapride	+378%	+210%	+509%			
AVP		+192%				
Vasopressin					+66%	+328%
Medical treatment						
Leuprolide acetate, monthly dose	3.75 mg IM	3.75 mg IM	3.6 mg IM	3.75 mg IM for 5 months changed to 11.25 mg IM every 3 months	3.75 mg IM for 4 months changed to 7.5 mg IM	3.75 mg IM for 5 months changed to 7.5 mg IM
Duration, months	24	12	10	40	12	10
Response	Clinical and biochemical control of hypercortisolism	Normal 24 h UFC maintained. No change in clinical symptoms	Partial resolution of symptoms and normalization of 24 h UFC initially. Escape after 8 months	Normalization of LDDST, decrease in midnight cortisol levels and restoration of suppressed ACTH values	Decrease in 1 mg DST and restoration of suppressed ACTH value	Decrease in 1 mg DST. Absence of restoration of ACTH suppressed value
*In vitro* study confirmation	No	No	Metoclopramide ACTH	No	No	No

* Peak value as a percentage of baseline value.

AVP, arginine vasopressin.

We describe two rare cases of MACS due to PBMAH associated with an aberrant cortisol response after LHRH stimulation and distinct treatment outcomes with leuprolide acetate.

MACS is associated with a significant burden of cardiometabolic comorbidities compared to non-functioning adrenal incidentalomas, including hypertension (RR: 1.24, 95% CI: 1.16–1.32), dyslipidemia (RR: 1.23, 95% CI: 1.13–1.34) and diabetes (RR: 1.44, 95% CI: 1.23–1.69). The risk of vertebral fractures is increased in studies using a serum cortisol cutoff of 83 nmol/L after 1 mg DST but not in studies using a cutoff of 50 nmol/L (RR: 1.08, 95% CI: 0.68–1.71) (2). Furthermore, the risk of all-cause mortality is significantly higher in patients with MACS. The risk of death is doubled in patients with a cortisol level between 83 and 137 nmol/L following 1 mg DST and tripled in those with a level above 138 nmol/L (9).

The management of MACS due to PBMAH remains controversial. According to the most recent guidelines on adrenal incidentalomas (2023), adrenalectomy is more likely to provide cardiometabolic benefit when (i) cortisol excess is greater (higher cortisol after 1 mg DST), (ii) relevant comorbidities are present (obesity, hypertension, diabetes mellitus, dyslipidemia, osteoporosis), and/or iii) additional biochemical abnormalities are found (low plasma ACTH, elevated 24 h UFC, or late-night salivary cortisol) (1, 2). Age, sex, overall health, and patient preference should also be considered when making treatment decisions. In most published cases of PBMAH, UA of the larger adrenal gland has been performed, given the correlation between cortisol excess and adrenal size (2). Bilateral adrenalectomy is rarely indicated in the absence of overt Cushing’s syndrome, as it is associated with higher morbidity and lifelong glucocorticoid and mineralocorticoid replacement therapy. Even though the initial remission rate is 97% after UA, there is a 20–70% risk of recurrent hypercortisolism, and about 33% of patients eventually require contralateral adrenalectomy (1).

In a subset of PBMAH patients, the identification of an abnormal *in vivo* response to aberrant stimuli, possibly reflecting aberrant hormone receptor expression, may offer the opportunity to control hypercortisolism with targeted medical therapy instead of adrenalectomy. A cortisol increase of >50% from baseline following stimulation is considered abnormal and may be treated with drugs targeting the specific aberrant receptor involved (4) However, clinically effective antagonists are not available for all aberrant receptors. To date, β-blockers have been used successfully in a few cases of catecholamine-dependent CS confirmed by exaggerated cortisol elevation after the upright posture test. Somatostatin analogues have been tried but provided only transient control of GIP-dependent CS. Leuprolide acetate has been used in cases of functionally abnormal LH/hCGR expression (4). Leuprolide acetate, a long-acting GnRH agonist, suppresses endogenous LH production and thereby limits stimulation of LHCGRs by its ligand. Reports of CS or MACS associated with aberrant LHCGR expression in adrenal tumors are rare: fewer than 20 cases are described in the literature, and only four of these patients were treated with leuprolide acetate (4).

Our first patient had an exaggerated cortisol response to stimulation with LHRH, vasopressin, and metoclopramide. Monthly leuprolide acetate 3.75 mg IM was initiated, as this was the dose used in three previously reported patients with PBMAH and an aberrant response to LHRH stimulation (5, 6, 7) (Table 4). A partial response was initially observed, and increasing the dose to 7.5 mg IM monthly led to a long-term improvement in hypercortisolism. This suggests that dose escalation to ensure adequate gonadotropin suppression may be attempted before considering surgery. Normalization of ACTH levels and reduction of 24 h UFC suggested treatment efficacy, even though cortisol levels after 1 mg DST did not fully normalize (20% reduction). In other reported cases, a biochemical improvement was demonstrated by normalization of 24 h UFC or morning cortisol, and in only one case did the patient normalize cortisol after LDDST (5, 6, 7). In our patient, leuprolide acetate stabilized worsening hypertension and dyslipidemia. In PBMAH, UA is associated with improvements in hypertension and hyperglycemia in 20 and 35% of patients, respectively (10). Thus, it is difficult to conclude that leuprolide acetate is inferior to UA for hypertension control in this case. No incident fragility fractures were observed. However, the follow-up remains too short to determine whether leuprolide acetate will provide durable control of comorbidities. Given the persistent abnormal 1 mg DST, UA was proposed, but due to advancing age, active smoking, and patient preference, she elected to continue medical therapy with leuprolide acetate.

In our second patient, morning cortisol after 1 mg DST decreased (from a mean of 102 nmol/L to 88 nmol/L), but ACTH levels remained suppressed despite treatment. We speculate that the lack of response may be due to the lower cortisol increment after gonadotropin stimulation (+58%) compared to our first patient (+142%) and the four previously reported cases (Table 4). This suggests that a greater cortisol response to gonadotropin stimulation may predict a better therapeutic response to leuprolide. In addition, decreased LH receptor expression over time may explain their minimal presence on pathology and the lack of clinical response. Furthermore, the stronger involvement of other aberrant stimuli, such as vasopressin and ACTH – both associated with marked cortisol increments in this patient, may have contributed to persistent hypercortisolism, as they were not targeted by treatment.

In conclusion, our cases demonstrate that systematic dynamic stimulation testing to assess cortisol responses to a variety of stimuli, especially LHRH, may guide targeted medical therapy as an alternative to adrenalectomy in selected PBMAH patients with MACS. A favorable response to treatment may delay or avoid surgery in certain cases. Our findings also suggest that a higher cortisol increment after LHRH stimulation may be associated with better response to leuprolide. This raises the question of whether a threshold higher than >50% cortisol elevation following dynamic stimulation should be considered when selecting patients for medical therapy as first-line treatment. Future studies are needed to better identify PBMAH patients with MACS or CS who will benefit most from targeted medical therapy with leuprolide acetate. In addition, the long-term cardiometabolic safety of such therapies must be established, particularly in patients who partially improve excessive cortisol secretion by normalizing ACTH and reducing 24 h UFC despite persistently abnormal 1 mg DST results. The true efficacy of this approach in reducing cardiometabolic burden and mortality risk in MACS remains uncertain. Larger-scale studies will be essential to validate these observations and to inform evidence-based treatment guidelines.

## Declaration of interest

MSt-J is a research investigator for Spruce Biosciences and a speaker for Recordati Rare Diseases and GlaxoSmithKline (GSK). LB-C is a speaker for Recordati Rare Diseases, Lilly, and Pfizer.

## Funding

This research did not receive any specific grant from any funding agency in the public, commercial or not-for-profit sector.

## Patient consent

Written informed consent was obtained from all patients for the publication of their clinical details and/or clinical images.

## Author contribution statement

All authors contributed equally to the conception, writing and editing the manuscript. M St-Jean is following the patient in the outpatient office. L Branchaud-Croisetière is an endocrinology fellow with a special interest in adrenal pathologies working with M St-Jean. M Maillet is an endocrinologist working with M St-Jean.
